# Analysis of Quantitative Phytochemical Content and Antioxidant Activity of Leaf, Stem, and Bark of *Gymnosporia senegalensis* (Lam.) Loes.

**DOI:** 10.3390/plants13111425

**Published:** 2024-05-21

**Authors:** Divya Jain, Mukesh Meena, Pracheta Janmeda, Chandra Shekhar Seth, Jaya Arora

**Affiliations:** 1 Department of Bioscience and Biotechnology, Banasthali Vidyapith, Tonk 304022, Rajasthan, India; divyajain31011996@gmail.com; 2 Department of Microbiology, School of Applied and Life Sciences, Uttaranchal University, Dehradun 248007, Uttarakhand, India; 3 Laboratory of Phytopathology and Microbial Biotechnology, Department of Botany, Mohanlal Sukhadia University, Udaipur 313001, Rajasthan, India; mukeshmeenamlsu@gmail.com; 4 Department of Botany, University of Delhi, New Delhi 110007, Delhi, India; csseth52@gmail.com; 5 Laboratory of Biomolecular Technology, Department of Botany, Mohanlal Sukhadia University, Udaipur 313001, Rajasthan, India; jaya890@gmail.com

**Keywords:** antioxidant activity, *Gymnosporia senegalensis*, medicinal plant, phytochemicals, quantitative estimation

## Abstract

To the best of our knowledge, there was no prior report providing valuable preliminary data through a demonstration of the quantitative phytochemical and antioxidant activity of *Gymnosporia senegalensis.* The total contents of phenols, flavonoid, flavanol, tannin, and saponin were evaluated from different fractions extracted from the leaf, stem, and bark of *G. senegalensis* by using standards such as gallic acid, quercetin, rutin, tannic acid, and saponin quillaja. The antioxidant potential was measured by 2,2-diphenyl-1-picrylhydrazyl (DPPH), hydrogen peroxide scavenging (H_2_O_2_), superoxide anion radical scavenging, metal chelating ferrous ion, ferric reducing antioxidant power (FRAP), and total antioxidant capacity (TAC). Data were subjected to half-inhibitory concentration (IC_50_) and one-way analysis of variance (ANOVA) at *p* < 0.05 as a significant value. The total phenol content was found to be highest in the chloroform extract of stem at 97.7 ± 0.02 mg GAE/g. The total flavonoid and flavonol contents in the aqueous extract were 97.1 ± 0.03 mg QE/g and 96.7 ± 0.07 mg RE/g, respectively. The total tannin content in the ethyl acetate extract of leaf was 97.5 ± 0.01 mg TAE/g, and the total saponin content in the methanol extract of stem was 79.1 ± 0.06 mg SQE/g. The antioxidant analysis indicated that IC_50_ and percentage (%) inhibition were dose-dependent and showed the highest antioxidant activity (40.9 ± 0.9 µg/mL) in methanol extract of leaf for DPPH, (88.8 ± 1.12 µg/mL) in the chloroform extract of stem for H_2_O_2_, (43.9 ± 0.15 µg/mL) in the aqueous extract of bark for superoxide anion radical scavenging activity, (26.9 ± 0.11 µg/mL) in the chloroform extract of leaf for the metal chelating ferrous ion activity, (7.55 ± 0.10 mg/mL) in the benzene extract of leaf for FRAP, and (2.97 ± 0.01 mg/mL) in the methanol extract of bark for TAC. These results show that *G. senegalensis* has great potential in antioxidant activities. The isolation and characterization of specific bioactive compounds and the in vivo applicability of such activity await further extensive studies for drug discovery and development.

## 1. Introduction

A comprehensive system of naturally occurring enzyme and non-enzyme-based antioxidant defense mechanisms protects the human body against the damaging effects of free radicals and other oxidants [1]. Free radicals are extremely reactive and are produced more when they interact with new compounds. Reactive oxygen species (ROS), such as hydroxyl radicals, hydrogen peroxide, and superoxide anion, probably play a role in the pathophysiology of various human diseases [2]. Free radicals and ROS are generally recognized as messengers in intracellular and intercellular signaling as well as damaging agents. Various diseases have been proposed to be treated using a variety of both natural and synthetic antioxidants, where the potential effects of oxygen free radicals have been implicated [3].

Replacing synthetic antioxidants like butylated hydroxyanisole (BHA) and butylated hydroxytoluene (BHT), which are known to have toxicity and health implications, with natural plant antioxidants is advisable [4]. In light of the significant advances in our understanding of the roles of ROS and antioxidants in living systems, a fundamental re-evaluation of assays for determining antioxidant activity is warranted. There is extensive scientific evidence demonstrating the crucial role of ROS in the development and progression of various diseases [5,6]. Therefore, it is essential to reassess antioxidant assays to better comprehend their effectiveness in mitigating ROS-related damage and disease processes. Modern scientific studies show that plants have contributed to the creation of natural antioxidant compositions for food, cosmetics, and other uses that improve human health [7,8]. The global population is increasingly attracted to embracing complementary and alternative medicine due to its low cost and fewer side effects [9,10,11].

In light of the significant advances in our understanding of the roles of ROS and antioxidants in living systems, a fundamental re-evaluation of assays for determining antioxidant activity is warranted. There is extensive scientific evidence demonstrating the crucial role of ROS in the development and progression of various diseases [12]. Therefore, it is essential to reassess antioxidant assays to better comprehend their effectiveness in mitigating ROS-related damage and disease processes [13,14]. Later in the discussion, we will explore the intricate interplay between ROS, antioxidants, and disease pathology to provide a clearer perspective on this matter.

*Gymnosporia senegalensis* (*G. senegalensis*) belongs to the Celastraceae family, which is well known for its various pharmacological properties. Traditional African healers used different parts to treat newborns suffering from fever, loss of appetite, snakebites, and general poor health, and to treat jaundice in adults. In India, it is used to treat several diseases like tertiary syphilis, diarrhea, dysmenorrhoea, stomach diseases, dyspepsia, eye infection, wounds, leprosy, malaria, chest pains, chronic illness, chest pains, rheumatism, etc. [15].

The plant extracts have been reported to possess multifaceted biological activities, including antibacterial, antifungal, cytotoxic, antimycobacterial, antioxidant, anti-inflammatory, antimalarial, antiedematogenic, and antinociceptive activities. The plants are globally distributed in the adjacent islands, Africa, India, Madagascar, Malaysia, Papua New Guinea, the Philippines, Pakistan, Queensland (Australia), Southern Spain, Sri Lanka, Taiwan, and the Polynesian Islands [16]. The plant thrives in regions with annual rainfall ranging up to 700 mm and typically attains a diameter of approximately 25–70 cm. It is predominantly found in tropical Africa and is prevalent in savannah areas, spanning from sea level to montane regions worldwide. With its potential to serve as a pioneer species in woodland restoration, especially in dryland regions like semi-arid areas and dune thickets, the plant holds significant promise [17]. Its leaves and roots are frequently traded in African markets, particularly in places like the Dakar market [18].

Various bioactive compounds were identified from the roots and stem of *G. senegalensis*, such as pristimerin, β-sitosterol, maytenoic acid, β-amyrin, and lupenone, for promoting the anti-inflammatory and anti-microbial potential of *G. senegalensis* [19]. Ndako et al. [20] revealed the presence of a few polar compounds in *G. senegalensis*, leaves such as isomintlactone, jacareubin, and iristectrorigenin. Whereas, Tatsimo [21] reported for the first time the occurrence of glycated cyanogenic derivatives and their carboxylic acid esters, which also contain two sterols, one monoglyceride, and fifteen phenolic bioactive constituents. Quercetin, flavanone, kaempferol, or epicatechin can be the main bioactive compounds.

Different parts (leaf, stem, and bark) of *G. senegalensis* were used to alleviate several human ailments such as diarrhea, wounds, dyspepsia, eye infection, malaria, dysmenorrhoea, rheumatism, chronic pain, and chest pains [22,23]. Several researchers have reported pharmacological activities like antimicrobial and anti-inflammatory activities [15,24,25,26]. Micro-anatomy is the initial set of pharmacognostic methods used to evaluate any fresh raw sample of medicinal plants.

The literature survey revealed that there was no information on the quantitative phytochemical estimation and in vitro antioxidant activities of various parts of *G. senegalensis*. Several assays were used to measure the antioxidant potential of the test samples, which had different chemical structures and polarities depending on the assay system in use. Depending on the plant biology system, the methods for the evaluation of antioxidant activities were divided into two categories. The first was quantitative phytochemical assays (total phenol, total flavonoid, total flavonol, total tannin, and total saponin), and the second was evaluation of the antioxidant activity of the 2,2-diphenyl-1-picrylhydrazyl (DPPH) radical scavenging assay, hydrogen peroxide scavenging assay, superoxide anion radical scavenging activity, metal chelating ferrous ions activity, ferric reducing antioxidant power assay (FRAP), and total antioxidant capacity (TAC) [27].

## 2. Results

### 2.1. Determination of Quantitative Phytochemical Assays

#### 2.1.1. Total Phenol Content (TPC)

The analysis of the results from the current study is listed in Table 1 and Figure S1A. The average TPC range was found to be 10.7–97.7 mg GAE/g in all extracts. The following is the ascending order of phenol content at a significant level (*p* < 0.05) in different fractions: MES (methanol extract of stem) < AEB (aqueous extract of bark) < AEL (aqueous extract of leaf) < EES (ethyl acetate extract of stem) < GA (gallic acid) < EEL (ethyl acetate extract of leaf) < CEB (chloroform extract of bark) < BEL (benzene extract of leaf) < PEB (petroleum ether extract of bark) < CEL (chloroform extract of leaf) < PES (petroleum ether extract of stem) < PEL (petroleum ether extract of leaf) < MEB (methanol extract of bark) < EEB (ethyl acetate extract of bark) < AES (aqueous extract of stem) < BES (benzene extract of stem) < BEB (benzene extract of bark) < MEL (methanol extract of leaf) < CES (chloroform extract of stem). The highest TPC was found to be 97.7 ± 0.02 mg GAE/g in the chloroform extract of stem, then 79.8 ± 0.02 mg GAE/g in the methanol extract of leaf, and 74.7 ± 0.02 mg GAE/g in the benzene extract of bark. This amount was compared with the gallic acid standard (40.7 ± 0.04) using a standard curve (y = 0.552x − 0.5105; R^2^ = 0.969).

#### 2.1.2. Total Flavonoid Content (TFC)

The present study shows a significant variation ranging from 19.0 ± 0.03 to 97.1 ± 0.03 (mg QE/g) in all extracts. The flavonoid content at a significant level (*p* < 0.05) in different fractions was in ascending order as follows: PEB < PES < PEL < EEB < AES < EES < MEB < BEL < Q (quercetin) < AEB < CEB < MES < CES < EEL < CEL < AES < BEB < BES < AEL. The highest TFC was found to be 97.1 ± 0.03 mg QE/g in the aqueous extract of leaf, 96.0 ± 0.01 and 70.7 ± 0.02 mg QE/g in the benzene extract of stem and bark, respectively. The linear equation for the quercetin (46.7 ± 0.01 mg QE/g) standard was (y = 0.2702x − 0.140; R^2^ = 0.956) from the straight line curve plotted between concentration and absorbance at 510 nm (Table 1 and Figure S1B).

#### 2.1.3. Total Flavonol Content

The total flavonol content revealed that it ranged from 12.9 ± 0.05 to 96.7 ± 0.07 (mg RE/g) in all extracts (Table 1 and Figure S1C). The following is the ascending order at a significant level (*p* < 0.05) in different fractions as follows: BES < BEL < AES < R (rutin) < PEL < EES < EEB < CES < PES < EEL < CEL < CEB < MES < MEL < AEB < MEB < BEB < PEB < AEL. The highest flavonol content was found in the aqueous extract of leaf at 96.7 ± 0.07 mg RE/g, then in the petroleum ether extract of bark at 59.7 ± 0.05 mg RE/g, and in the methanol extract of stem at 47.8 ± 0.08 mg RE/g from the standard curve of different concentrations of rutin (39.4 ± 0.05 mg RE/g) having a linear equation of (y = 0.7325 − 1.0609; R^2^ = 0.915).

#### 2.1.4. Total Tannin Content

The total tannin content revealed that it ranged from 35.6 ± 0.04 to 97.5 ± 0.01 (mg TAE/g) in all extracts (Table 1 and Figure S1D). The ascending order of tannin content at a significant level (*p* < 0.05) in different fractions was as follows: AEB < TA (tannic acid) < PEL < MEL < CEB < PEB < BEB < MES < BES < PES < MEB < AEL < BEL < CES < AES < EES < CEL < EEL. The highest amount of tannins was present in the ethyl acetate extract of leaf and stem at 97.5 ± 0.01 mg TAE/g and 71.8 ± 0.01 mg TAE/g, then in the benzene extract of bark at 48.8 ± 0.06 mg TAE/g. This quantitative estimation was performed using a standard curve of tannic acid (38.7 ± 0.02 mg TAE/g). The linear equation came to be (y = 0.6842x − 0.9692; R^2^ = 0.9226).

#### 2.1.5. Total Saponin Content

The total saponin content ranged from 11.2 ± 0.04 to 79.1 ± 0.06 (mg SQ/g) in all extracts (Table 1 and Figure S1E). The ascending order of the saponin content at a significant level (*p* < 0.05) in different fractions was as follows: AEB < CES < CEB < PES < BEB < CEL < MEB < BEL < MEL < PEL < SQ (saponin quillaja) < BES < ESS < AEL < PEB < EEL < AES < EES < MES. Quantitative estimation of saponin was performed by plotting a standard curve of saponin quillaja. The highest saponin content was found to be 79.1 ± 0.06 mg SQ/g in the methanol extract of stem, then in the ethyl acetate extracts of bark and leaf at 63.6 ± 0.07 and 56.3 ± 0.03 mg SQ/g, respectively. This quantitative estimation was performed using a standard curve of saponin (40.9 ± 0.02 mg SQE/g). The linear equation came to be (y = 0.7104x − 1.0009; R^2^ = 0.917).

### 2.2. Evaluation of Antioxidant Activity

#### 2.2.1. 2,2-Diphenyl-1-picrylhydrazyl (DPPH) Assay

DPPH is sensitive to the detection of active compounds at micromolar concentrations, so this test is frequently used to detect antioxidant activity. The DPPH reduction ability listed in Table 2 and Figure S2A shows that the extract has a standard accordance scavenging property. The ascending order of the DPPH IC_50_ values at a significant level of (*p* < 0.05) was as follows: AC < EES < MES < EEL < CES < PEL < AES < BES < BEL < CEL < CEB < AEB < BEB < EEB < MEB < PEB < LEL < PES < MEL. The results indicated that the ethyl acetate extract of the stem shows the lowest IC_50_ at 1.91 ± 0.01 and the methanol extract of the leaf shows the highest IC_50_ at 40.9 ± 0.9 µg/mL among all the fractions of *G. senegalensis*.

#### 2.2.2. Hydrogen Peroxide Scavenging Assay

The result of the reduction in H_2_O_2_ scavenging is listed in Table 2 and Figure S2B. The scavenging capability for H_2_O_2_ of various extracts at a significant level (*p* < 0.05) was in ascending order as follows: EEL < CEL < AEB < EEB < MEB < AC < AES < PEL < EES < BES < MEL < BEB < PES < CEB < BEL < PEB < MES < AEL < CES. It showed a very effective reduction at 0.11 ± 0.01 µg/mL in the leaf ethyl acetate, then in the leaf chloroform extract at 0.62 ± 0.02 µg/mL, the bark aqueous extract at 2.81 ± 0.03 µg/mL, followed by maximum scavenging in the chloroform extract of the stem at 88.8 ± 1.12 µg/mL as determined by the IC_50_ value.

#### 2.2.3. Superoxide Anion Radical Scavenging Assay

The IC_50_ of the superoxide anion radical scavenging assay at a significant level (*p* < 0.05) was in ascending order as follows: CEB < BEB < BES < EEB < EEL < PEL < PES < CES < CEL < PEB < BHA < BEL < MEB < MEL < AEL < EES < MES < AES < AEB. Therefore, the decrease in absorption of antioxidants indicates the consumption of superoxide anion in the reaction mixture. In the current study, superoxide anion levels generated in vitro were found to be lowest in the chloroform extract of the bark at 0.16 ± 0.01 µg/mL, followed by benzene extract of bark and stem at 0.72 ± 0.01 and 0.76 ± 0.01 µg/mL, respectively. The highest IC_50_ was found in the aqueous extract of bark at 43.9 ± 0.15 µg/mL (Table 2 and Figure S2C).

#### 2.2.4. Metal Chelating Ferrous Ions Assay

The data obtained from the current study revealed that all *G. senegalensis* extracts showed an impactful capacity for iron-chelation (Table 2 and Figure S2D). The IC_50_ of the metal chelating ferrous ions assay at a significant level (*p* < 0.05) was in ascending order as follows: BES < CES < AEB < MES < BEB < PES < BEB < EES < AES < PEL < PEB < EEB < EEL < AC < MEL < AEL < BEL < CEB < CEL. This indicates that the benzene extract of stem at 0.30 ± 0.01 µg/mL showed the lowest IC_50_ followed by the aqueous extract of bark at 0.91 ± 0.03 µg/mL, and the chloroform extract of leaf at 26.9 ± 0.11 µg/mL showed the highest IC_50_ to chelate iron.

#### 2.2.5. Ferric Reducing Antioxidant Power (FRAP) Assay

The percentage (%) inhibition of the FRAP assay at a significant level (*p* < 0.05) was in ascending order as follows: PEB < PEL < CEB < PES < EEB < AEB < BHT < CES < BEB < BES < AEL < CEL < MEB < AES < MEL < EEL < EES < MES < BEL. The antioxidant potentials of various extracts were found to be lowest in the petroleum ether extract of bark and leaf at 0.71 ± 0.01 and 0.73 ± 0.06 mg/mL and highest in the benzene extract of leaf at 7.55 ± 0.10 mg/mL, respectively (Table 2 and Figure S3A).

#### 2.2.6. Total Antioxidant Activity (TAC)

The TAC value of *G. senegalensis* extracts was found to be considerably higher as compared to the standard, which is ascorbic acid (Table 2 and Figure S3B). The % inhibition of TAC at a significant level (*p* < 0.05) was in ascending order as follows: EEL < PES < AEL < AEB < PEB < BEB < BES < CES < MEL < AES < PEL < EEB < BEL < AC < MES < CEB < EES < CEL < MEB. The antioxidant potentials of the extracts of various parts of *G. senegalensis* were found to be lowest in the ethyl acetate extract of leaf at 0.15 ± 0.01 mg/mL, followed by the petroleum ether extract of stem at 0.19 ± 0.01 mg/mL, the aqueous extract of leaf at 0.27 ± 0.01 mg/mL, and higher in the methanol extract of bark at 2.97 ± 0.01 mg/mL, respectively.

## 3. Discussion

Natural antioxidants and their health benefits have recently attracted a lot of attention. To observe how effective an antioxidant is in preventing the oxidation of biological molecules, a huge number of research studies on the protection of biological targets by antioxidants have been published so far. This depends on various types of secondary metabolites that possess flavonoids, saponins, tannins, and phenols [28,29,30]. Flavonoids are a diverse group of phytonutrients that belong to the larger class of compounds known as polyphenols. Flavonoids are further classified into several subgroups based on their chemical structure. Some common types and groups of flavonoids include the following: flavones, flavonols, flavanones, isoflavones, anthocyanins, and proanthocyanidins. These phytochemicals have multiple therapeutic activities that include antioxidant, antimicrobial, anti-cancerous, and anti-inflammatory activities. The presence of reductants, which break the free radical chain by donating an atom of hydrogen or by preventing the formation of peroxide, is directly related to their reducing ability [31].

In the literature, it is widely acknowledged that oxidative stress plays a role in the development of chronic diseases, including cancer and cardiovascular disease, and the ability of antioxidants to reduce various toxic and harmful effects [32,33]. Therefore, it is necessary to explore new antioxidant sources. Various plant extracts extracted using solvents based on their polarity index were used and evaluated in the current study, which demonstrated the variability in antioxidant characteristics. In the current study, we report phytochemical analysis and antioxidant assays on different parts (leaf, stem, and bark) of *G. senegalensis* for the first time. Conclusively, the antioxidant role played by various secondary metabolites such as phenols, flavonoids, tannins, etc., reported in various studies showed ROS inhibition, lipid peroxidation, platelet aggregation, membrane stabilization, restoration of vascular function, and antioxidant enzymes [34,35].

### 3.1. Quantitative Estimation

#### 3.1.1. Total Phenol Content

Phenol compounds are important compounds that exist in plants. Because of their hydroxyl groups, they have the ability to scavenge. They play a vital role in quenching singlet and triplet oxygen, adsorbing or decomposing peroxides, and neutralizing free radicals. Phenol compounds such as tannins, flavonoids, and phenolic acid possess various biological activities such as anti-carcinogenic, anti-bacterial, and anti-inflammatory activities. Multiple studies have proposed that medicinal plants that have high polyphenol contents may be the reason for their antioxidant capacity and other biological effects [36,37,38]. This is used in the defense process against ROS in such a way that it helps the plants by causing molecular damage to pests, microorganisms, herbivores, and insects [39,40].

In the present study, gallic acid is used as a standard and is one of the most abundant phenolic acids, which acts as a useful antioxidant agent and has various health-promoting effects [41]. The results show that the level of phenol compounds evaluated in the extracts of *G. senegalensis* was relatively significant. It was reasonable to assess the total amount of plant phenol compounds in experimental plant extracts because they are one of the main categories of chemicals that act as principal antioxidants or free radical terminators.

#### 3.1.2. Total Flavonoid and Flavonols Content

In plants, flavonoids help combat oxidative stress. However, quercetin showed the strongest ability to chelate metal ions (iron and copper). The possible mechanisms of their action are considered to be their ability to inhibit several enzymes and produce free radicals, such as lipoxygenase, xanthine oxidase (XO), phosphoinositide 3-kinase, and cyclo-oxygenase (COX). Various studies have found that flavonoids can protect against a variety of diseases [42,43]. Compared to the findings of the literature for various extracts of plant products [44], our results suggested that phenol compounds, flavonoids, and flavonols may be the main contributors to antioxidant activity.

#### 3.1.3. Total Tannin Content

On the basis of their chemical composition and biological properties, tannins are divided into two groups: hydrolyzable tannins and condensed tannins. Both are able to bind firmly with specific kinds of proteins and slow the process of their digestion [45,46]. Recent studies have investigated the significant antioxidant and antibacterial properties of tannins. Due to their healing properties, tannins can be used to cure a variety of diseases, such as cardiovascular diseases, cancer, diarrhea, gastritis, inflammatory bowel disease (IBD), diabetes, dermatitis, etc., and improve human health as they possess anti-carcinogenic and anti-mutagenic properties [47,48].

#### 3.1.4. Total Saponin Content

Saponin is considered as a part of the defense mechanism of the plants. In one of the studies, the role of saponin was identified as a compound that causes membrane permeability, resulting in cell leakage and cytotoxic, and antibacterial effects [49]. These results also favor earlier research that included plants from the Celastraceae family. In this approach, Kpoyizoun et al. [50] showed a higher level of phenol, flavonoid, and tannin content from the hydro-ethanolic extract of *Maytenus senegalensis*. Different techniques are needed to evaluate the antioxidant activity of *G. senegalensis* due to its complicated chemical composition. Therefore, six complementary approaches were used in the current investigation to assess the capacity to eliminate free radicals.

#### 3.1.5. DPPH

**Figure 1:**
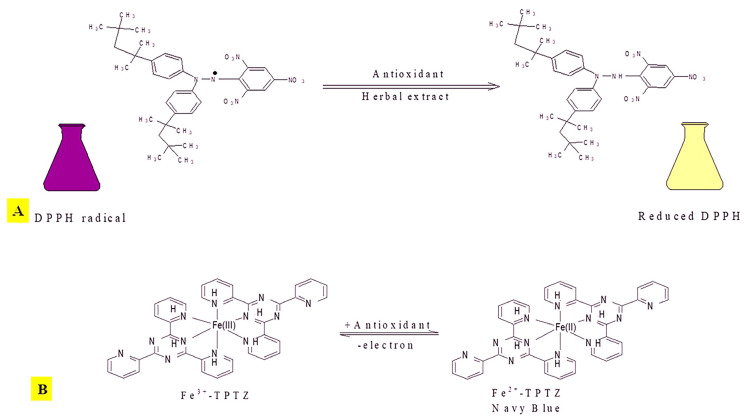
Mechanism of action. (**A**) DPPH with an antioxidant having transferable hydrogen radical. (**B**) Formation of Fe (II) from Fe (III) complex by antioxidants.

DPPH is a widely used free radical for evaluating the antioxidant activity of natural antioxidants through a non-enzymatic reaction. This free radical assay is enhanced by decreasing the number of DPPH radicals in the presence of a hydrogen-donating antioxidant. By hydrogen or electron donation, the color of the solution changes in this process from purple to yellow, and the amount of hydrogen that can be donated by the extracts has the effect of scavenging free radicals (Figure 1A). All studied extracts (petroleum ether, benzene, chloroform, ethyl acetate, methanol, and aqueous) had lower DPPH radical scavenging capacities as compared with ascorbic acid. This result could be due to extraction efficiency, synergistic interactions with other compounds present in the extracts, or concentration and composition variations. As a result, compounds that can lower DPPH could be classified as antioxidants and radical scavengers [51,52,53].

In the current study, the leaf methanol extract had a substantially higher IC_50_ than all other fractions evaluated due to the presence of various bioactive compounds, i.e., alkaloids, flavonoids, phenols, etc. This indicates that the plant extracts contain phytochemical elements that can donate hydrogen to a free radical to scavenge potential damage. Furthermore, the concentration of each extract of phenol compounds and its ability to scavenge free radicals were closely associated. Our previous study suggested that alkaloids, glycosides, cyanogenetics, and anthraquinone glycosides were found in higher amounts in the methanol extract of leaves than in the chloroform extract of *G. senegalensis* as compared to stem and bark extracts [54]. In our previous study, we revealed the presence of the structures of four bioactive compounds, i.e., tetratetracontana derivative, β-carotene, amyrin, and terpineol. In our previous study, GC-MS analysis revealed a diverse array of chemical entities with both high and low molecular weights, encompassing volatile and essential oils, monoterpenoids, tetraterpenoids, carotenoids, terpenoids, triterpenes, and nortriterpenes, each present in varying quantities. These findings suggest that *G. senegalensis* harbors a rich assortment of bioactive compounds with significant biological and pharmacological relevance. A dose of crude methanolic extract and a polar fraction of *G. senegalensis* up to 5000 mg/kg were administered for 24 h. The dose of 1000 mg/kg was considered the maximum tolerated dose of both extracts [20].

Variations in the plant growth environment or the extraction method could be the cause of the slight difference. Interestingly, *G. senegalensis* extracts of different parts exhibited stronger scavenging activity than standard ascorbic acid. Standard ascorbic acid functions as a chain-breaking antioxidant that harms the body’s ability to create intracellular compounds by promoting the production of free radicals. It includes dentine, collagen, and bone matrix [55].

#### 3.1.6. Hydrogen Peroxide Scavenging Assay

Humans are indirectly exposed to H_2_O_2_ through the environment at a rate of nearly 0.28 mg/day, with the majority of their consumption coming from leaf crops. It may enter the human body through eye and dermal contact and the inhalation of vapor or mist. Because of its high reactivity, it can react with any biomolecule. It quickly breaks down into oxygen and water, which could result in hydroxyl radicals that start lipid peroxidation and cell death, and can damage DNA [56]. Therefore, the elimination of hydroxyl radicals is crucial for the protection of the living system. The bioactive compounds responsible for the hydrogen peroxide elimination capacity can often be correlated with the polarity of the solvent used for extraction. Due to the presence of phenolic groups, which can donate electrons to hydrogen peroxide and neutralize it in water, extracts from various portions of *G. senegalensis* effectively scavenged H_2_O_2_ [57,58,59].

#### 3.1.7. Superoxide Anion Scavenging Assay

Superoxide anions are weak oxidants that generate extremely potent and dangerous hydroxyl radicals and singlet oxygen, which are the oxidative stress-causing agents [60,61]. They can decrease the activity of various antioxidant defense enzymes, such as catalase, glutathione peroxidase, and energy metabolism schemes such as NADH dehydrogenase. These radicals are considered a significant biological source of ROS and a reductant for transition metals due to the presence of phenolic compounds [62]. Butylated hydroxyanisole (BHA) showed concentration-dependent scavenging of superoxide radicals at all concentrations.

According to the results presented, the ethyl acetate extract of leaf (EEL), chloroform extract of leaf (CEL), and aqueous extract of stem (AEB) were identified as the most significant in terms of their ability to eliminate superoxide anions. Superoxide anion elimination capacity is often associated with antioxidant compounds that possess moderate to high polarity. Ethyl acetate and chloroform extracts are typically considered to be of moderate polarity, while the aqueous extract is highly polar. The bioactive compounds responsible for eliminating superoxide anions may include various antioxidants such as phenolic compounds, flavonoids, tannins, and other polar compounds. These compounds are known for their ability to scavenge reactive oxygen species, including superoxide anions, due to their electron-donating properties and ability to stabilize free radicals [63,64]. The results of our study revealed that *G. senegalensis* has antioxidant potential due to its ability to effectively scavenge superoxide radicals.

#### 3.1.8. Metal Chelating Assay

The reduction power of the *G. senegalensis* extracts is due to chelating activity, which is needed to compete with ferrozine for the ferrous ions, and accelerate the rate of lipid peroxidation through the Fenton reaction [56]. According to current results, many plant extracts exhibit significant metal chelating activity due to the presence of flavonoids and phenol compounds. However, metal chelating activity is often associated with compounds that possess moderate to high polarity, such as flavonoids, phenolic compounds, and other polyphenols [65,66]. These compounds have functional groups like hydroxyl (-OH) and carbonyl (-c=o) groups, which are capable of forming complexes with metal ions, thereby preventing them from participating in oxidative processes.

#### 3.1.9. FRAP

Reducing the power activity of various plant extracts is a determining factor that depends on their electron-donating capacity and corresponds to their antioxidant activity in a concentration-dependent manner due to the presence of phenols, tannins, and flavonoids. The Fe^3+^-TPTZ [2,4,6-Tris(2-pyridyl)-s-triazine] complex is reduced by the test reducing power capability, which forms ferrous sulfate in the presence of a reductant (antioxidants) in the solution (Figure 1B). Our findings indicate that the extracts work as an electron donor, a reliable indicator of antioxidant activity, and can act as both primary and secondary antioxidants [67]. The higher antioxidant potential (polyphenols, carotenoids, flavonoids, glycosides, cyanogenetics, etc.) observed in the benzene extract of the leaf compared to the petroleum ether extract of the bark and leaf can be attributed to differences in solvent extraction efficiency and the concentration of antioxidant bioactive compounds present in each extract.

#### 3.1.10. TAC

The ability of extracts to prevent deoxyribose from being degenerated by the hydroxyl radicals produced in the reaction mixture serves as an indicator of their hydroxyl radical scavenging capacity. Other studies have reported that various plants in the Celastraceae family have antioxidant activity. Weli et al. [68] reported that *Maytenus dhofarenis* bark contained a high concentration of antioxidant compounds. According to the results of this study, *G. senegalensis* can serve as a good source of antioxidants for pharmaceutical preparations and applications that enable the cellular system to cope with oxidative stress and alleviate the toxic effects of the administrative compound. With the knowledge of different radical scavenging properties, it becomes easy to establish a relationship between the endogenous and exogenous antioxidant systems, which is important for the naturally derived herbal drugs’ functioning and bioavailability in the body [69,70,71,72,73].

## 4. Materials and Methods

### 4.1. Literature Search

To search the literature related to the relation between the phytochemical content and antioxidant activity of *Gymnosporia senegalensis*, a systematic search of more than 100 articles on Embase, Science Direct, Web of Science, Google Scholar, PubMed, and Scopus was done with the help of scientific keywords such as “phytochemical analysis”, “antioxidant activity”, “*Gymnosporia senegalensis*”, “quantitative estimation”, “Celastraceae”, “DPPH”, etc.

### 4.2. Collection of Plant Materials

The different parts (leaf, stem, and bark) of *G. senegalensis* were collected from the nearby areas of Banasthali, Tehsil Newai, Rajasthan, India, at latitude and longitude of 26.40211, 75.87648 in January 2022. According to the International Plant Names Index, the species plantarum number is 17:541 1893. The plant parts were identified and authenticated by a renowned taxonomist from the Patanjali Herbal Research Department, Haridwar, India. For further reference, an herbarium specimen with the serial number PRFH/005 was deposited at the Patanjali Research Foundation Herbarium.

### 4.3. Chemicals, Reagents, and Extract Preparation

The reagents and chemicals used in the present study were of analytical grade and procured from reputable companies including Himedia (Mumbai, India), CDH (New Delhi, India), Merck (Darmstadt, Germany), and SRL (Mumbai, India), ensuring the highest available purity and reliability. Moreover, glassware and apparatus were purchased from the companies of Borosil (Mumbai, India), Tarsons (Kolkata, India), and Riviera (Mumbai, India). The fresh leaves, stems, and barks of *G. senegalensis* were thoroughly washed and then shade dried at room temperature for approximately one month. Subsequently, these samples were finely ground into a homogeneous powder using an electric grinder (Panasonic MKGW200, Kadoma, Japan). The resulting powder was sieved through a 40-mesh sieve to ensure uniformity and stored at room temperature for subsequent analysis. Various sensory parameters, such as color, texture, and odor, were examined through organoleptic studies on the fresh samples to determine their purity and identity.

Fifty grams of finely grinded dried powder from each part of the plant were used to prepare sequential extracts by using a hot continuous soxhlation extraction method with different solvents (petroleum ether, benzene, chloroform, ethyl acetate, methanol, and aqueous) in ascending order of polarity ranging from non-polar to polar. The soxhlet mixture was filtered using muslin cloth, and concentrated under vacuum pressure using a rotary evaporator at the boiling point of each solvent. Subsequently, the dried extracts were stored at 4 °C in an airtight container for further use [74].

### 4.4. Quantitative Phytochemical Assays

#### 4.4.1. Total Phenol Content

The determination of the total phenol content (TPC) was evaluated using the Folin–Ciocalteu (FC) reagent method with a slight modification [75]. Two ml of various samples was added to a volumetric flask containing 5 mL of methanol, 1 mL of a 1:10 dilution, FC reagent solution, and 500 µL of Na_2_CO_3_. After 90 min of incubation at 25 °C, absorbance (spectrophotometer; ELOCO double beam SL 210 UV-VIS) was recorded at 765 nm. Gallic acid was used as a standard and expressed as gallic acid equivalents (mg GAE/g).

#### 4.4.2. Total Flavonoid Content

The total flavonoid content (TFC) was measured with a colorimetric assay using aluminum chloride (AlCl_3_) as described by Ordoñez et al. [76]. One mg of each plant extract was diluted with 500 µL of distilled water and mixed with 0.3 mL of sodium carbonate (Na_2_CO_3_). All test tubes were incubated for 5 min at 25 °C. This was followed by the addition of 300 µL of aluminium trichloride solution (AlCl_3_) and 1 mL of sodium hydroxide (NaOH) and mixed well. The absorbance was noted at 510 nm. The flavonoid content was expressed as a calibration curve of equivalent quercetin (mg QE/g).

#### 4.4.3. Total Flavonol Content

The total flavonol content in each plant extract was estimated by the aluminium trichloride (AlCl_3_) colorimetric method of Kumaran and Karunakaran [31]. Briefly, 2 mL of various samples, 1000 µL of AlCl_3_ solution, and sodium acetate solution were added. The change in reaction mixture color was evaluated after incubation for 2.5 h at 20 °C, and absorbance was recorded at 440 nm. The total flavonol content was expressed as equivalent rutin (mg RE/g).

#### 4.4.4. Total Tannin Content

The total tannin content was estimated according to the method reported by Patel et al. [58] with slight modifications. Briefly, 2 mL of various extract samples was added with 500 µL methanol and each Folin–Denis reagent solution. The reaction mixture was incubated for 10 min at room temperature, and the absorbance was recorded at 700 nm. Tannin content was expressed as mg of equivalent tannic acid per gram of dry weight (mg TAE/g).

#### 4.4.5. Total Saponin Content

The estimation of the total saponin content was carried out using the vanillin-sulfuric acid colorimetric reaction method [77]. In 2 mL of various samples, 50 µL of vanillin reagent in methanol and 2.5 mL of sulfuric acid (H_2_SO_4_) were added. This solution was properly mixed and kept in a water bath for 15 min at 60 °C. Then it was cooled in ice-cold water, and the absorbance was taken at 560 nm. The values were expressed as equivalent saponin quillaja (mg SQE/g).

### 4.5. Evaluation of In Vitro Antioxidant Activity

#### 4.5.1. 2,2-Diphenyl-2-picrylhydrazyl (DPPH) Radical Scavenging Assay

This is the most commonly documented assay to evaluate the antioxidant activity of different compounds present in many plant systems. It is determined according to the method of Braca et al. [78]. Briefly, a stock methanolic solution of DPPH (0.03 mM) was prepared and kept in the dark. To 2 mL of the sample having different concentrations (200–1000 µg/mL), 1000 µL of DPPH solution was added and vortexed. The reaction mixture was incubated in a dark place at room temperature for 30 min, and then absorbance was recorded at 517 nm using ascorbic acid as a standard. The scavenging activity (SA) was estimated using the following equation:

SA (%) = (Acontrol − Asample/Acontrol) × 100

where A_control_ is the absorbance of the control and A_sample_ is the absorbance of the tested sample.

#### 4.5.2. Scavenging Activity Hydrogen Peroxide (H_2_O_2_)

The ability of extracts to scavenge H_2_O_2_ was determined using the method of Ruch et al. [57]. The H_2_O_2_ solution was prepared by mixing 50 mM phosphate buffer with 40 mM H_2_O_2._ For the reaction mixture, aliquots of extracted samples having different concentrations (200–1000 µg/mL) were prepared by adding 0.2 mL of distilled water and 0.6 mL of H_2_O_2_ solution. The test tubes were then shaken well and allowed to stand for 15 min. The absorbance was measured at 230 nm against phosphate buffer as blank and ascorbic acid as positive control. The hydroxyl radical scavenging ratio was calculated using the following formula:

Percentage (%) inhibition of H_2_O_2_ = [(*A_i_* − *A_t_*)/*A_i_*] × 100

where *A_t_* = Absorbance of the sample; *A_i_* = Absorbance of the control; and where the control was the phosphate buffer with H_2_O_2_.

#### 4.5.3. Superoxide Anion Radical Scavenging Activity

The assay was determined by using reduction of nitroblue tetrazolium (NBT) in the nicotinamide adenine dinucleotide-nitroblue tetrazolium-phenazine methosulfate (NADH-NBT-PMS) system in the presence of light [79]. The reaction mixture contains 1 mL each of the test sample, NBT (78 µmol), and NADH (468 µmol). This reaction was further followed by the addition of 0.4 mL of PMS (60 µmol, pH 7.4). The test tubes were incubated for 5 min at room temperature. The absorbance was estimated at 560 nm against phosphate buffer as blank and BHA as standard. Scavenging activity % was followed by the following formula:

Scavenging % = [(*A_i_* − *A_t_*)/*A_i_*] × 100

where *A_t_* = Absorbance of the sample; *A_i_* = Absorbance of control.

#### 4.5.4. Ability to Chelate Metal-Ferrous Ions Activity

The Fe^2+^ chelating ability of test samples was measured using the ferrous iron–ferrozine complex method of Dinis et al. [80]. The test sample extracts were dissolved in methanol to prepare different concentrations of test samples (200–1000 µg/mL). Then 50 µL of ferrous chloride and 200 μL of ferrozine were added to the test samples. The reaction mixture was then vigorously shaken and incubated for 10 min at room temperature. Absorbance was recorded against methanol as a blank and ethylenediaminetetraacetic acid (EDTA) as a standard at 562 nm. The amount of metal ion chelated by 1 g of chelating agent is indicated by the chelating value. Ferrous chloride (FeCl_2_) and ferrozine complex-forming molecules were eliminated from the control. The following formula was used to calculate the capacity of the extracts to chelate ferrous ions:

Chelating ferrous ion (%) = [(A_0_ − A_1_) A_0_] × 100

where A_0_ is the absorbance of the control, and A_1_ is the absorbance in the presence of samples of extracts or standards. The control does not contain FeCl_2_ and ferrozine, complex formation molecules.

#### 4.5.5. Determination of Ferric Reducing Antioxidant Power (FRAP) Assay

The FRAP assay was carried out according to the method of Yen and Chain [67] with slight modifications. Briefly, the FRAP reagent was freshly prepared by mixing sodium acetate buffer, TPTZ solution in HCl, and FeCl_3_ solution in 10:1:1 (*v*/*v*) proportions. The FRAP reagent was warmed to 37 °C before use. The test sample (2 mL) was allowed to react for 30 min in a water bath in the dark with 2 mL of the FRAP reagent solution. The absorbance of the reaction mixture was then measured at 593 nm. The standard curve was obtained using a sulfuric acid (FeSO_4_) solution. The results were expressed as mg/mL.

#### 4.5.6. Total Antioxidant Capacity

Total antioxidant capacity (TAC) was determined using the phosphomolybdenum method described by Prieto et al. [81]. One ml of sulfuric acid, ammonium molybdate, and sodium phosphate was mixed with 1 mL of test solution. The tubes were properly mixed and incubated at 95 °C in a hot water bath for 60 min, and then cooled to room temperature. Then its absorbance was measured at 765 nm against sodium phosphate buffer as a blank and ascorbic acid as a standard. TAC was expressed as an ascorbic acid equivalent (mg ACE/g).

### 4.6. Statistical Analysis

The experimental results were expressed as mean ± standard deviation (SD) in triplicate. The half-inhibitory concentration (IC_50_) of the extracts was calculated by plotting the concentration of the plant extract against the corresponding percentage of radical scavenging activity and fitting a linear model from this equation, and the antioxidant value was determined to scavenge 50% of free radicals in a reaction mixture. In a linear regression of the plotted graph, the X-axis represented the concentration of test samples, and the Y-axis represented the mean percentage of inhibitory ability of three replicates. The data were subjected to a one-way analysis of variance (ANOVA), and the differences between samples was determined by Tukey’s multiple comparison tests (*p* < 0.05) using the SPSS version 16.0 (Statistical Program for Social Sciences) program for Windows (IBM, Armonk, NY, USA). Microsoft Office 2010 was used for data analysis.

## 5. Conclusions

Discovering antioxidant molecules from natural sources that are pharmacologically active and have almost no side effects is currently of greater interest on a global scale. The increased use of various pollutants, pesticides, chemicals, alcohol intake, smoking, and some synthetic drugs increases the risk of diseases induced by free radicals. Therefore, it is now necessary to investigate, identify, and understand our traditional therapeutic understanding and the plant sources to give it the place it deserves in the fight against oxidative stress due to the presence of various phytochemicals that can help in curing various human ailments. We are aware that no prior reports on comparative preliminary results of the quantitative and antioxidant activity of *G. senegalensis* were provided. In summary, studies investigating the phytochemical composition and biological activities of *G. senegalensis* plant extracts have the potential to make significant contributions to various fields, including drug discovery, nutraceutical development, environmental conservation, public health, and natural product research.

## Figures and Tables

**Table 1:** Total phenol, flavonoid, flavonol, tannin, and saponin content in different parts (leaf, stem, and bark) of *G. senegalensis*.

Parts	Extracts and Standards	Total Phenol (mg GAE/g)	Total Flavonoid (mg QE/g)	Total Flavonol (mg RE/g)	Total Tannin (mg TAE/g)	Total Saponin (mg SQE/g)
Leaf	PEL	50.7 ± 0.06	37.7 ± 0.08	39.5 ± 0.03	39.2 ± 0.16	40.1 ± 0.05
	BEL	44.6 ± 0.01	45.1 ± 0.03	35.7 ± 0.40	57.8 ± 0.04	39.1 ± 0.08
	CEL	44.9 ± 0.20	62.2 ± 0.01	46.2 ± 0.01	80.7 ± 0.13	36.6 ± 0.03 ^a^
	EEL	41.8 ± 0.03	53.3 ± 0.08	45.2 ± 0.07	97.5 ± 0.01 ^a^	56.3 ± 0.03
	MEL	79.8 ± 0.02 ^a^	39.0 ± 0.02	49.8 ± 0.04	42.8 ± 0.07	39.6 ± 0.04
	AEL	39.5 ± 0.09	97.1 ± 0.03 ^a^	96.7 ± 0.07 ^a^	55.8 ± 0.05	49.9 ± 0.08
Stem	PES	47.5 ± 0.03	20.0 ± 0.07	44.7 ± 0.05	52.9 ± 0.44	15.1 ± 0.01
	BES	59.1 ± 0.08	96.0 ± 0.01 ^a^	12.1 ± 0.05	51.4 ± 0.02	42.2 ± 0.01
	CES	97.7 ± 0.02 ^a^	52.1 ± 0.03	44.6 ± 0.07	63.9 ± 0.02	12.2 ± 0.09
	EES	40.1 ± 0.05	39.3 ± 0.08	41.4 ± 0.04	71.8 ± 0.01	48.2 ± 0.01
	MES	10.7 ± 0.01 ^a^	51.1 ± 0.05	47.8 ± 0.80 ^a^	50.3 ± 0.12	79.1 ± 0.06 ^a^
	AES	56.1 ± 0.07	62.9 ± 0.01	37.2 ± 0.03	65.4 ± 0.02 ^a^	57.8 ± 0.09
Bark	PEB	44.8 ± 0.07	19.0 ± 0.03	59.7 ± 0.05 ^a^	45.7 ± 0.07	51.7 ± 0.07
	BEB	74.7 ± 0.02 ^a^	70.7 ± 0.02 ^a^	58.0 ± 0.01	48.8 ± 0.06	24.7 ± 0.01
	CEB	42.7 ± 0.06	48.1 ± 0.02	46.2 ± 0.01	43.2 ± 0.01	13.5 ± 0.01
	EEB	52.7 ± 0.02	37.9 ± 0.06	41.9 ± 0.02	39.1 ± 0.04	63.6 ± 0.07 ^a^
	MEB	50.0 ± 0.03	39.8 ± 0.07	57.0 ± 0.01	53.4 ± 0.03 ^a^	38.3 ± 0.01
	AEB	38.9 ± 0.05	47.3 ± 0.01	56.2 ± 0.01	35.6 ± 0.04	11.2 ± 0.04
S	GA	40.7 ± 0.04	-	-	-	-
	Q	-	46.7 ± 0.01	-	-	-
	R	-	-	39.4 ± 0.05	-	-
	TA	-	-	-	38.6 ± 0.02	-
	SQ	-	-	-	-	40.9 ± 0.02

The values represent the means of the triplicates ± SD. ^a^ (*p* < 0.05) compared to standard. GA: gallic acid; Q: quercetin; R: rutin; TA: tannic acid; SQ: saponin quillaja; PEL: petroleum ether extract of leaf; BEL: benzene extract of leaf; CEL: chloroform extract of leaf; EEL: ethyl acetate extract of leaf; MEL: methanol extract of leaf; AEL: aqueous extract of leaf; PES: petroleum ether extract of stem; BES: benzene extract of stem; CES: chloroform extract of stem; EES: ethyl acetate extract of stem; MES: methanol extract of stem; AES: aqueous extract of stem; PEB: petroleum ether extract of bark; BEB: benzene extract of bark; CEB: chloroform extract of bark; EEB: ethyl acetate extract of bark; MEB: methanol extract of bark; AEB: aqueous extract of bark; S: standard.

**Table 2:** IC_50_ and percentage (%) inhibition of different extracts and standards.

Parts	Extracts and Standards	Half-Inhibitory Concentration (IC_50_)				Percentage (%) Inhibition	
		DPPH (µg/mL)	Hydrogen Peroxide (µg/mL)	Superoxide (µg/mL)	Metal Chelation (µg/mL)	FRAP (mg/mL)	TAC (mg/mL)
Leaf	PEL	3.31 ± 0.01	29.9 ± 0.11	1.23 ± 0.01	3.13 ± 0.04	0.73 ± 0.06	0.72 ± 0.03
	BEL	3.45 ± 0.02	51.1 ± 0.19	3.99 ± 0.03	14.4 ± 0.06	7.55 ± 0.10 ^a^	1.21 ± 0.02
	CEL	3.63 ± 0.01	0.62 ± 0.02	3.13 ± 0.01	26.9 ± 0.11 ^a^	5.12 ± 0.01	1.75 ± 0.09 ^a^
	EEL	2.89 ± 0.01	0.11 ± 0.01	0.93 ± 0.01	4.60 ± 0.02	6.39 ± 0.05	0.15 ± 0.01
	MEL	40.9 ± 0.9	35.7 ± 0.13	10.8 ± 0.04	5.07 ± 0.02	5.99 ± 0.05	0.50 ± 0.01
	AEL	14.9 ± 0.07	63.3 ± 0.21 ^a^	17.7 ± 0.07 ^a^	12.5 ± 0.06	3.55 ± 0.01	0.27 ± 0.01
Stem	PES	20.1 ± 0.05	43.9 ± 0.14	2.54 ± 0.02	1.39 ± 0.03	0.85 ± 0.01	0.19 ± 0.01
	BES	3.41 ± 0.01	32.4 ± 0.11	0.76 ± 0.01	0.30 ± 0.01	3.12 ± 0.01	0.42 ± 0.01
	CES	3.22 ± 0.02	88.8 ± 1.12	2.56 ± 0.01	0.33 ± 0.01	2.65 ± 0.01	0.44 ± 0.08
	EES	1.91 ± 0.01	30.5 ± 0.11	19.5 ± 0.09	1.87 ± 0.02 ^a^	6.43 ± 0.04	1.51 ± 0.09 ^a^
	MES	2.04 ± 0.01	58.1 ± 0.15	22.8 ± 0.09	1.02 ± 0.02	6.69 ± 0.02 ^a^	1.43 ± 0.02
	AES	3.39 ± 0.02	26.8 ± 0.09 b	24.2 ± 0.11 ^a^	2.04 ± 0.02	5.58 ± 0.02	0.55 ± 0.06
Bark	PEB	11.1 ± 0.02	56.9 ± 0.09 ^a^	3.15 ± 0.02	3.25 ± 0.03	0.71 ± 0.01	0.35 ± 0.04
	BEB	4.53 ± 0.01	42.4 ± 0.08	0.72 ± 0.01	1.17 ± 0.03	2.66 ± 0.01	0.38 ± 0.01
	CEB	4.04 ± 0.01	44.1 ± 0.08	0.16 ± 0.01	15.7 ± 0.06 ^a^	0.76 ± 0.01	1.43 ± 0.08
	EEB	8.34 ± 0.04	19.0 ± 0.07	0.82 ± 0.02	4.37 ± 0.03	1.42 ± 0.02	1.18 ± 0.05
	MEB	8.67 ± 0.04	21.3 ± 0.08	8.37 ± 0.02	1.51 ± 0.02	5.62 ± 0.01 ^a^	2.97 ± 0.01 ^a^
	AEB	4.61 ± 0.01	2.81 ± 0.03	43.9 ± 0.15 ^a^	0.91 ± 0.03	1.85 ± 0.01	0.31 ± 0.13
S	AC	64.8 ± 0.12	22.4 ± 0.09	-	4.83 ± 0.01	-	1.27 ± 0.05
	BHA	-	-	3.92 ± 0.01	-	-	-
	BHT	-	-	-	-	2.37 ± 0.01	-

The values represent the means of the triplicates ± SD. ^a^ (*p* < 0.05) compared to standard. AC: ascorbic acid; BHA: butylated hydroxyanisole; FRAP: ferrous reducing power assay; TAC: total antioxidant capacity; BHT: butylated hydroxytoluene; PEL: petroleum ether extract of leaf; BEL: benzene extract of leaf; CEL: chloroform extract of leaf; EEL: ethyl acetate extract of leaf; MEL: methanol extract of leaf; AEL: aqueous extract of leaf; PES: petroleum ether extract of stem; BES: benzene extract of stem; CES: chloroform extract of stem; EES: ethyl acetate extract of stem; MES: methanol extract of stem; AES: aqueous extract of stem; PEB: petroleum ether extract of bark; BEB: benzene extract of bark; CEB: chloroform extract of bark; EEB: ethyl acetate extract of bark; MEB: methanol extract of bark; AEB: aqueous extract of bark; S: standard.

## Data Availability

The datasets used and/or analyzed during the current study are available from the corresponding author on reasonable request. The data also presented in this study are available in Supplementary Materials.

## Supplementary Materials

The following supporting information can be downloaded at: https://www.mdpi.com/article/10.3390/plants13111425/s1, Figure S1. Quantitative analysis of various parts of *G. senegalensis*. Figure S2. IC_50_ values of various parts of *G. senegalensis*. Figure S3. Percent inhibition of various parts of *G. senegalensis*.

## Abbreviations

DPPH, 2,2-diphenyl-1-picrylhydrazyl; H_2_O_2_, hydrogen peroxide; FRAP, ferric reducing antioxidant power; TAC, total antioxidant capacity; IC_50_, half-inhibitory concentration; ROS, reactive oxygen species; TPC, total phenol content; TFC, total flavonoid content; XO, xanthine oxidase; COX, cyclo-oxygenase; IBD, inflammatory bowel disease; FC, Folin–Ciocalteu; AlCl_3_, aluminium trichloride solution; NaOH, sodium hydroxide; SA, scavenging activity; µg, microgram; ml, milliliter; NBT, nitroblue tetrazolium; NADH, nicotinamide adenine dinucleotide; PMS, phenazine methosulfate; FeCl_2_, ferrous chloride; FeSO_4_, sulfuric acid; TAC, total antioxidant capacity; EDTA, ethylenediaminetetraacetic acid; SD, standard deviation; ANOVA, analysis of variance; SPSS, Statistical program for social sciences; TPTZ, 2,4,6-Tris(2-pyridyl)-s-triazine.
