# Supplementary material for: Analysis of Quantitative Phytochemical Content and Antioxidant Activity of Leaf, Stem, and Bark of Gymnosporia senegalensis (Lam.) Loes
Source: Plants (Basel). 2024 May 21;13(11):1425. doi: 10.3390/plants13111425 (PMC11174610; doi:10.3390/plants13111425)
Supplement: Supplementary file 1 [file plants-13-01425-s001.zip › plants-3000004-supplementary.pdf]

plants

Article

Analysis of Quantitative Phytochemical Content and Antioxidant Activity of Leaf, Stem, and Bark of *Gymnosporia senegalensis* (Lam.) Loes.

Divya Jain [1,2], Mukesh Meena [3], Pracheta Janmeda [1,*], Chandra Shekhar Seth [4] and Jaya Arora [5]

[1] Department of Bioscience and Biotechnology, Banasthali Vidyapith, Tonk 304022, Rajasthan, India; divyajain31011996@gmail.com
[2] Department of Microbiology, School of Applied and Life Sciences, Uttaranchal University, Dehradun 248007, Uttarakhand, India
[3] Laboratory of Phytopathology and Microbial Biotechnology, Department of Botany, Mohanlal Sukhadia University, Udaipur 313001, Rajasthan, India; mukeshmeenamlsu@gmail.com
[4] Department of Botany, University of Delhi, New Delhi 110007, Delhi, India; csseth52@gmail.com
[5] Laboratory of Biomolecular Technology, Department of Botany, Mohanlal Sukhadia University, Udaipur 313001, Rajasthan, India; jaya890@gmail.com
* Correspondence: pracheta@banasthali.in

Supplementary Material

Supplementary Figure S1

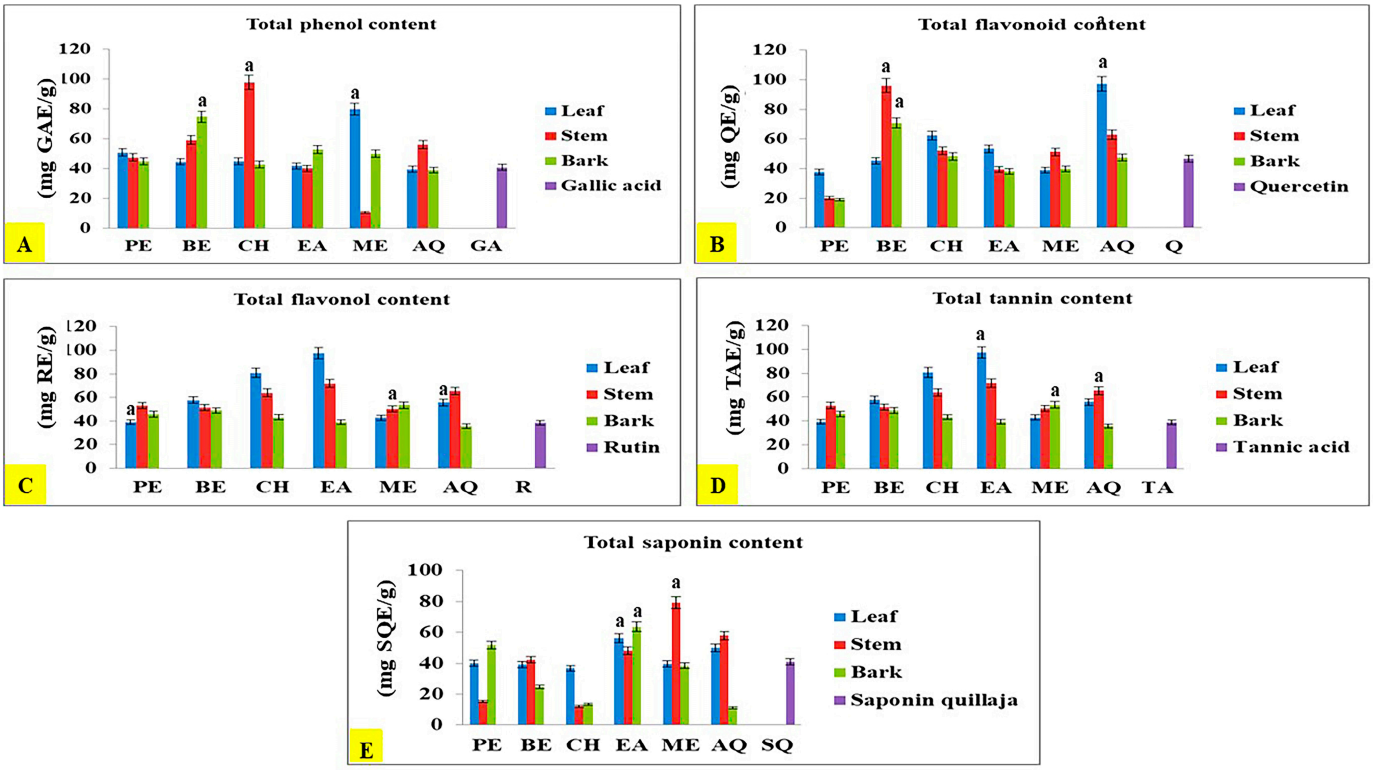

Figure S1. Quantitative analysis of various parts of *G. senegalensis*. (A) total phenol content (B) total flavonoid content (C) total flavonol content (D) total tannin content (E) total saponin content. PE: petroleum ether, BE: benzene, CH: chloroform, EA: ethyl acetate, ME: methanol, AQ: aqueous, GA: gallic acid, Q: quercetin, SQ: saponin quillaja, TA: tannin acid. R: rutin

Supplementary Figure S2

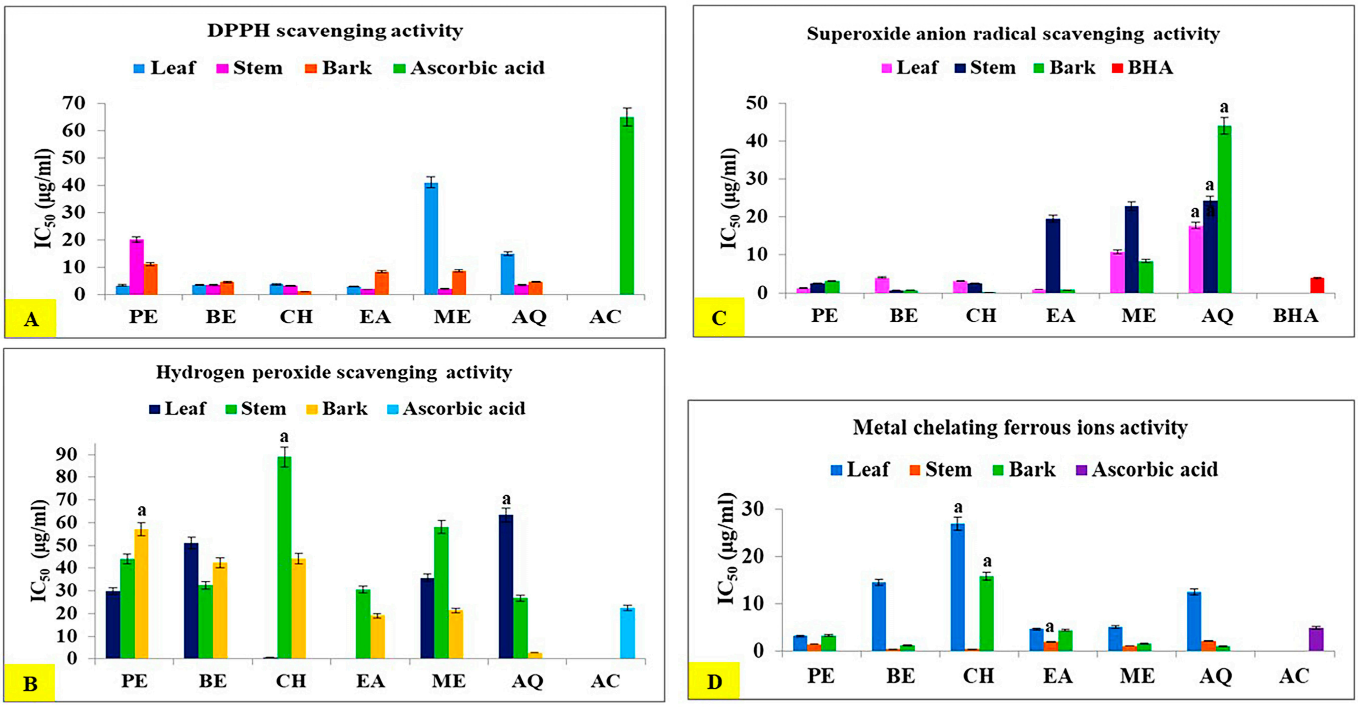

Figure S2. IC50 values of various parts of *G. senegalensis*. (A) DPPH scavenging activity (B) hydrogen peroxide scavenging assay (C) superoxide anion radical scavenging assay (D) metal chelating ferrous ions assay. PE: petroleum ether, BE: benzene, CH: chloroform, EA: ethyl acetate, ME: methanol, AQ: aqueous, AC: ascorbic acid, BHA: butylated hydroxyanisole

Supplementary Figure S3

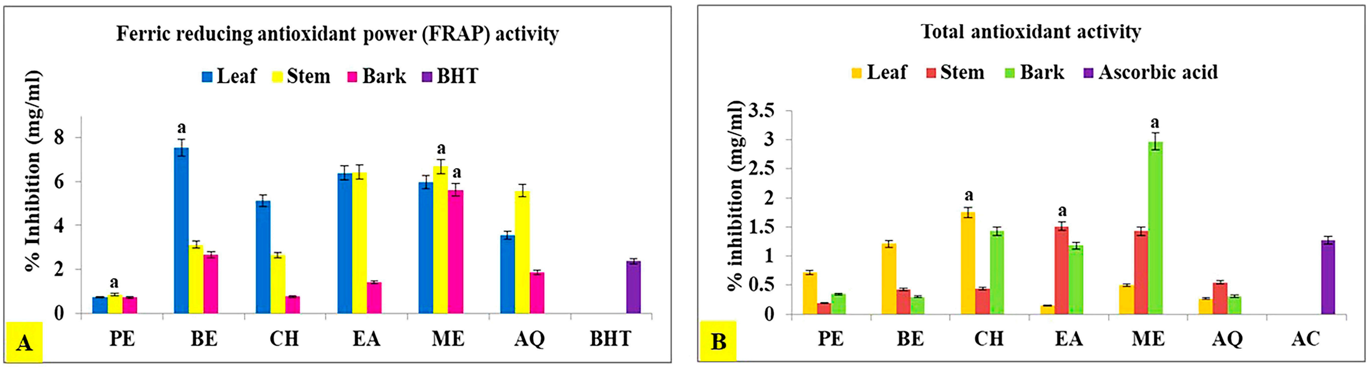

Figure S3. Percent inhibition of various parts of *G. senegalensis*. (A) Ferric reducing antioxidant power (FRAP) assay (B) total antioxidant activity. PE: petroleum ether, BE: benzene, CH: chloroform, EA: ethyl acetate, ME: methanol, AQ: aqueous, BHT: butylated hydroxytoluene, AC: ascorbic acid
